# Elexacaftor – Tezacaftor – Ivacaftor treatment improves systemic infection parameters and *Pseudomonas aeruginosa* colonization rate in patients with cystic fibrosis a monocentric observational study

**DOI:** 10.1016/j.heliyon.2023.e15756

**Published:** 2023-04-24

**Authors:** Alexander Schnell, Hannah Hober, Natalie Kaiser, Renate Ruppel, Annika Geppert, Christina Tremel, Julia Sobel, Erika Plattner, Joachim Woelfle, André Hoerning

**Affiliations:** a Department of Pediatrics and Adolescent Medicine, University Hospital Erlangen, Friedrich-Alexander-University Erlangen-Nuremberg, Germany; b First Department of Medicine, University Hospital Erlangen, Friedrich-Alexander-University Erlangen-Nuremberg, Germany

**Keywords:** Cystic fibrosis, CFTR modulator, Clinical data, Inflammation markers, Elexacaftor, Tezacaftor, Ivacaftor, ETI, Kaftrio

## Abstract

**Background & aims:**

The CFTR-modulating therapy Elexaftor – Tezacaftor - Ivacaftor (ETI) has been widely prescribed since its approval in 2020 in the European Union. The aim of this study was to methodically evaluate the effects of an ETI treatment on clinical, biochemical data and Pseudomonas colonization in order to demonstrate its efficacy.

**Methods:**

This prospective monocentric study comprised 69 patients diagnosed with cystic fibrosis aged at least 12 years and treated with ETI between September 2020 and November 2021. Clinical and laboratory data of each patient and study visit were collected before and after 24 weeks of ETI treatment. Follow-up status of *Pseudomonas aeruginosa* (PsA) colonization was assessed after one year of therapy by regularly determined sputum or throat swab samples.

**Results:**

Marked improvements biochemical markers of systemic inflammation as white blood cell count, levels of immunoglobulins A, G and M and albumin within 24 weeks of therapy were observed. ETI treatment proved to be effective as seen by amelioration of lung function and sweat chloride concentration. Assessment of PsA colonization status revealed a conversion from a positive to negative detection in 36% of the cases after one year of therapy.

**Conclusions:**

ETI treatment effectively improves systemic inflammation parameters and shows promising results in PsA status conversion.

## Highlights

• 24-week treatment with ETI improves biochemical markers of inflammation and as well as status of Pseudomonas colonization.

## 1 Introduction

Cystic fibrosis is a life-threatening disease due to mutations within *Cystic Fibrosis Transmembrane Conductance Regulator* gene (CFTR) that encodes for the equally named, cAMP-regulated chloride channel [1]. With one in 3.300–4800 neonates affected, cystic fibrosis is the most common autosomal-recessive hereditary disorder in Germany [2,3].

Cystic fibrosis may lead to organ manifestations like exocrine pancreas insufficiency and diabetes, liver cirrhosis as well as intestinal obstruction, but the most severe complication is pulmonary disease due to recurring infections (e.g., *Pseudomonas aeruginosa*) and subsequent deterioration of lung function. The subsequent chronic failure in pulmonary function still represents the most common cause for patients' death in almost 50% of the cases [4]. In the past decades, improvements in therapy of cystic fibrosis have clearly ameliorated as median age of survival increased from four to five years in the 1950’s to 50 years in Europe nowadays [[4], [5], [6]].

Until recently, the medical regimen was still limited to symptomatic treatment like inhalation of hypertonic saline for better secretolysis, substitution of pancreatic enzymes and antimicrobial therapy [7]. However, the development of a new class of drugs, the so-called CFTR modulators, has set a new milestone in the treatment of cystic fibrosis since Ivacaftor, a CFTR potentiator, proved to be very efficacious in patients carrying a G551D gating mutation and was subsequently introduced in 2012 [8]. Since then, research on this class of drugs has been further advanced and culminated with the approval of the triple combination therapy Elexacaftor – Tezacaftor – Ivacaftor (ETI) for patients with F508del homozygous mutations or F508del heterozygous and a minimal function mutation (Tricafta®: US 03/2020, Kaftrio®: EU 09/2020) in 2020.

Elexacaftor and Tezacaftor are two CFTR correctors that facilitate CFTR processing in the ER, whereas Ivacaftor as a potentiator prolongs CFTR channel opening and therefore (at least partially) restores CFTR function. In the phase III studies reported by Middleton and Heijerman [9,10], this drug combination led to an average improvement of lung function (FEV_1_) by 14% as well as a clear reduction of sweat chloride concentration. These impressive results are further underlined by our own clinical observations in the daily practice as quality of patients' life seemed to rise whereas exacerbation and hospitalization rate rather declined.

To demonstrate the drugs' efficacy in a real world setting and application in the daily clinical routine, we initiated this monocentric prospective cohort study that enables the objective collection and evaluation of clinical and biochemical data on the effectiveness of ETI therapy derived from a cohort comprising 69 participants. The aim of this study was to methodically evaluate the effects of an ETI treatment on clinical and laboratory data used as routine screening parameters in clinical care during an observational period of six months after treatment initiation.

## 2 Material and methods

### 2.1 Patients

The study cohort comprised 69 patients diagnosed with cystic fibrosis aged at least 12 years, between September 2020 and November 2021.

The study was approved by the local ethics' committee (vote number 20-485-B), registered (clinicaltrials.gov ID NCT05576324), and performed in accordance with the Declaration of Helsinki. Informed consent was obtained of all study participants or their legal custodians prior to study inclusion. Study visits up to four weeks prior and after six months of ETI therapy were arranged on the occasion of regular, usually quarterly appointments in the CF outpatient clinic. Here, actual body measures, body plethysmography, sweat chloride as well as biochemical parameters were assessed.

At study initiation, patients who were already under ETI treatment, so the study visit prior to ETI treatment was analyzed retrospectively. Therefore, some of the parameters are missing for these patients prior to ETI therapy. For the study visit after 6 months of therapy, all patients received the standardized assessment of the above-mentioned parameters. Patients that did not have started ETI therapy prior to study inclusion received standardized assessment for both study visits.

Exclusion parameters were defined as pregnancy at initiation and during ETI treatment as well as systemic intake of corticosteroids. Patients that did not take ETI until the follow-up time point after six months were also excluded from the study. A sample size calculation was not performed prior to study initiation due to the observational character of the study.

### 2.2 Clinical data

Clinical and laboratory data of each patient and study visit were collected from the hospital’s electronic health files and recorded pseudonymized. The patients' demographic data are summarized in Table 1.

**Table 1:** Baseline characteristics of the study cohort prior to ETI.

Age	27.56 yrs (SD ± 12.29 yrs; range: 12–56 yrs)	
	<18 yrs	17 (Mean 14.8 yrs, SD ± 1.5 yrs)
	>18 yrs	52 (Mean 31.4 yrs, SD ± 11.1 yrs)
Sex	f	27 (39%)
	m	42 (61%)
Mutation	F508del homozygous	39 (57%)
	F508del heterozygous	29 (42%)
	other	1 (1%)
CF-related diabetes	yes	24 (35%)
	no	45 (65%)
Pretreatment with CFTR modulator other than ETI	Iva	8 (11%)
	Teza + Iva	17 (25%)
	Luma + Iva	3 (4%)
FEV1	69.3% (SD ± 21.3%; range 27–108%)	
	>80%	25 (Mean 92.1%, SD ± 8.2%)
	<80%	44 (Mean 56.3%, SD ± 14.0%)
*Pseudomonas aeruginosa* colonization	yes	33 (48%)
	no	36 (52%)

Clinical data included sex, gender, age, mutation and body mass index (BMI, kg/m2) for adults as well as and BMI Z-score for juveniles. For lung function tests, forced vital capacity (FVC, %), forced expiratory volume in 1 s (FEV1, % of nominal value defined by Global Lung Initiative Reference Values [11]), maximal expiratory flow at 25% of FVC (MEF25, %), effective airway resistance (sReff, %), residual volume (RV, %), total lung capacity (TLC, %) ratio of RV and TLC (RV/TLC, %) were analyzed.

Laboratory data contained white blood cell count (WBC, x103/μl), HbA1c (%), plasma creatinine (mg/dl), total bilirubin (mg/dl), direct bilirubin (mg/dl), gamma-glutamyltransferase (γ-GT, U/l), alanin-aminotransferase (ALT, U/l), aspartate-aminotransferase (AST, U/l), cholinesterase (CHE, kU/l), glutamate-dehydrogenase (GLDH, U/l), C-reactive protein (CRP, mg/l), albumin (g/l), immunoglobulin G, M, A (g/l), E (kU/l).

For the evaluation of PsA colonization status, microbiological testing (sputum or deep throat swabs) was provided by patients with CF routinely four to six times per year in accordance with the current CF guidelines. A singular positive result in either of both diagnostic modalities was sufficient to label a patient PsA positive. Chronic PsA colonization was defined by three or more positive results out of at least six tests within one year.

For the PsA status conversion, at least 3 negative results within a period of at least a year after initiation of ETI treatment was necessary in accordance with the german CF care guidelines [12].

### 2.3 Statistical analysis

Statistical analysis was performed using GraphPad Prism software (Version 9, Graph Pad, San Diego, USA). Differences of clinical and laboratory data between baseline and after six months of ETI therapy were analyzed using multiple paired t-testing as the longitudinal data are compared from the same individual. Subcategory analyses for age (pediatric versus adult), LFT (FEV_1_, >80% versus <80%), *Pseudomonas aeruginosa* colonization (baseline positive versus baseline negative), previous medication with CFTR modulators other than ETI (CFTR modulator pretreatment versus CFTR modulator naïve) and genotype (F508del homozygous versus F508del heterozygous) were performed by multiple unpaired t-testing regarding differences between both time points.

Analysis of the association of the *Pseudomonas aeruginosa* colonization status and its alteration over time during the treatment with Kaftrio was assessed according to the guidelines after one year of therapy and was tested using Fisher’s exact test.

## 3 Results

### 3.1 Demographic characteristics of the study population

**Fig. 1:**
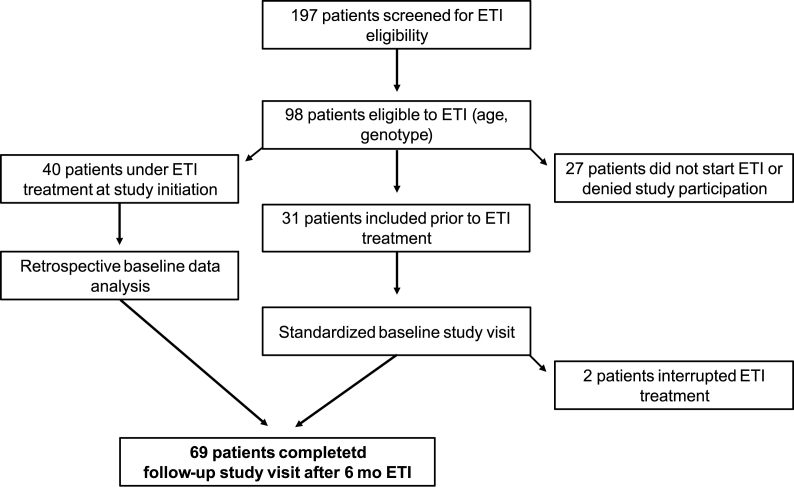
Study flow.

For this study, 98 patients were screened. Baseline characteristics were analyzed retrospectively for 40 patients, whereas 31 patients could be included prospectively. 2/31 did not reach study end point due to non-compliance. In total, 69 patients completed the follow-up study visit after six months of ETI therapy. The study flow is summarized in Fig. 1. The average follow-up interval was 6.37 months. The demographic data on our study cohort are summarized in Table 1. Subcategory analyses are also indicated in Table 1. Notably, one patient (m, 15 years) could be included taking ETI in off-label use though not having a F508del mutation because ETI treatment had been shown to be efficacious for his specific mutation combination (R347P/M1101K) [13,14].

### 3.2 ETI treatment effectively improves lung function parameters

Analysis of the most routinely used lung function parameters as determined by body plethysmography demonstrated improvements of almost all relevant markers except for TLC under ETI therapy (Table 2). As expected, there was a clear augmentation of FEV_1_ for the entire study cohort with an overall increase of 10 %-points, but also MEF25 as a parameter for obstruction of smaller airways and FVC proved to be ameliorated by 15.5 and 5.7 %-points, respectively. In contrast to that, we could observe very distinct decreases in the values for airway resistance (sR_eff_, −36.8 %-points), RV (−17.7 %-points) and ratio of RV/TLC (−0.2) over the course of ETI therapy. Moreover, patients' BMI and BMI z-Score showed an evident increase, too.

**Table 2:** Lung function parameters and BMI at baseline and after six months ETI treatment. BMI values are indicated for all adult patients whereas BMI z-Score is provided for juveniles.

Baseline						Follow-up							
	**Mean [t0]**	**SD [t0]**	**95% CI [t0] lower limit**	**95% CI [t0] upper limit**	**n**	**Mean [t6]**	**SD [t6]**	**95% CI [t6] lower limit**	**95% CI [t6] upper limit**	**n**	**Difference**	**SE**	P-**value**
FVC (%)	86.1	15.2	82.5	89.8	69	91.6	17.6	87.3	95.9	67	5.7	1.57	<0.001
FEV 1 (%)	69.3	21.3	64.2	74.4	69	79.4	21.0	74.3	84.5	67	10.0	1.31	<0.001
FEV1/FVC	0.8	0.2	0.8	0.8	69	0.9	0.4	0.8	1.0	67	0.1	0.05	0.015
MEF 25 (%)	44.2	33.3	36.2	52.2	69	60.0	42.1	49.7	70.3	67	15.5	2.45	<0.001
sReff (%)	184.6	93.2	161.5	207.7	65	144.7	92.5	121.8	167.6	65	−36.8	7.92	<0.001
RV (%)	142.9	51.9	130.1	155.6	66	123.3	34.5	114.7	131.8	65	−17.7	4.66	<0.001
TLC (%)	105.2	13.1	102.0	108.4	66	105.5	12.1	102.5	108.5	65	0.6	0.95	0.125
RV/TLC	1.4	0.5	1.3	1.5	66	1.2	0.3	1.1	1.2	65	−0.2	0.05	<0.001
BMI	21.7	2.8	20.9	22.5	52	22.4	2.8	21.6	23.2	51	0.7	0.39	<0.001
BMI z-Score	−0.4	1.2	−1.0	0.3	17	0.1	1.0	−0.4	0.6	17	0.5	0.001	0,027

With respect to subcategory analyses, the group with a FEV_1_ restriction <80% displayed a much more pronounced change of FEV_1_ (13.4 vs 7.4 %-points, q ≤ 0.01), RV (28.5 vs −7.6 %-points; q ≤ 0.01) and RV/TLC (−0.34 vs −0.08, q ≤ 0.01) in comparison to patients with FEV_1_ > 80%, respectively. Furthermore, patients receiving a CFTR modulator other than ETI prior to the initiated Kaftrio® treatment displayed a tendency to a rather minor increase of FEV_1_ than patients naïve to a CFTR modulator. However, this effect did not reach the set threshold of statistical significance (7.1 vs 12.2 %-points, q = 0.15). Yet, the change of sR_eff_ proved to be a significantly for this subanalysis: Whereas CFTR-modulator pretreated patients showed almost no difference of sR_eff_ values (+8.52 %-points) before and under treatment, CFTR-modulator naïve patients had a marked decrease of sR_eff_ values on average (−52.0 %-points; q = 0.02). Subcategory analysis regarding age, *Pseudomonas aeruginos*a colonization status and genotype yielded no relevant results.

### 3.3 ETI therapy ameliorates biochemical parameters of patients with CF

Regarding biochemical parameters (Table 3), we also observed clear signs of a restored CFTR channel function under ETI treatment as assessed by sweat chloride as a direct surrogate parameter for CFTR function. In our patient cohort, we detected a highly significant reduction of sweat chloride. Moreover, the average levels of serum sodium showed a trend for an increase in the therapy course, whereas serum chloride did not change.

**Table 3:** Laboratory parameters at baseline and after six months ETI treatment.

Baseline						Follow-up							
	**Mean [t0]**	**SD [t0]**	**95% CI [t0] lower limit**	**95% CI [t0] upper limit**	**n**	**Mean [t6]**	**SD [t6]**	**95% CI [t6] lower limit**	**95% CI [t6] upper limit**	**n**	**Difference**	**SE**	P-**value**
WBC (x10^3/μl)	8.9	3.2	8.1	9.6	69	6.4	2.1	5.9	6.9	68	−2.5	0.36	<0.001
HbA1c (%)	6.0	1.0	5.7	6.2	61	5.6	0.9	5.4	5.9	66	−0.3	0.08	0.0006
Kreatinin (mg/dl)	0.7	0.2	0.7	0.8	68	0.9	0.9	0.7	1.1	69	0.2	0.05	0.009
Bilirubin (total, mg/dl)	0.6	0.5	0.5	0.7	58	0.9	0.6	0.8	1.1	69	0.3	0.02	<0.001
Bilirubin (direct, mg/dl)	0.3	0.4	0.2	0.4	52	0.4	0.3	0.3	0.5	69	0.1	1.58	<0.001
AST (U/l)	25.8	9.9	23.4	28.2	69	28.2	16.1	24.3	32.1	69	2.4	2.37	0.13
ALT (U/l)	27.0	15.3	23.3	30.6	69	31.7	21.7	26.5	36.9	69	4.7	0.04	0.047
γGT (U/l)	31.9	40.9	22.0	41.8	68	35.6	57.0	21.9	49.3	69	3.6	2.6	0.177
CHE (U/l)	7.7	2.6	7.1	8.4	62	7.4	1.8	7.0	7.8	69	−0.3	0.2	0.131
GLDH (U/l)	6.8	12.0	3.2	10.5	43	5.7	6.0	4.3	7.2	67	−1.4	1.85	0.459
CRP (mg/l)	7.9	10.1	5.3	10.5	59	8.6	13.4	4.1	13.2	35	0.0	2.86	0.987
Albumin (g/l)	41.6	3.8	40.6	42.6	61	44.5	3.2	43.7	45.3	69	3.1	0.53	<0.001
IgG (g/l)	13.9	4.0	12.9	14.9	67	11.4	2.9	10.8	12.1	69	−2.5	0.27	<0.001
IgM (g/l)	1.1	0.7	0.9	1.4	26	1.2	0.7	1.0	1.3	69	−0.1	0.04	0.0589
IgE (kU/l)	187.3	340.1	99.5	275.2	60	186.9	495.9	67.7	306.0	69	18.0	50.17	0.717
IgA (g/l)	3.0	1.8	2.5	3.4	55	2.3	1.3	2.0	2.6	68	−0.6	0.09	<0.001
Albumin/Globulin ratio	3.3	1.1	3.0	3.5	59	4.2	1.2	3.9	4.5	68	0.9	0.09	<0.001
Sweat chloride (mmol/l)	95.3	21.6	88.7	102.0	44	48.6	20.1	39.7	52.6	41	−46.7	3.17	<0.001
Sodium (mmol/l)	138.1	2.1	137.7	138.7	69	139.8	2.0	139.3	140.3	67	1.6	0.32	<0.001
Chloride (mmol/l)	102.5	12.0	99.5	105.6	63	106.8	18.9	103.1	112.3	68	4.3	2.6	0.104

In the context of ETI treatment, special attention needs to be paid on a potential elevation of liver enzymes as indicated in the Summary of Product Characteristics. In our study cohort, persistently elevated liver enzymes were present in 15/69 patients (about 22%), especially with respect to ALT. Out of these 15 patients, two patients displayed more than a threefold elevation in comparison to baseline. In both patients, therapy could be resumed after normalization of serum transaminases during a short ETI-free interval with a reduced dosage. Moreover, a slight but highly significant increase in the levels of total and direct bilirubin could also be observed. In addition to that, γ-GT as another cholestatic parameter tendentially increased under ETI, however this finding did not reach the level of statistical significance. With respect to hepatic protein synthesis capacity, a pronounced increase of serum albumin levels could be observed in the longitudinal study course. Other hepatic enzymes like CHE or GLDH did not show any difference. Also, renal function parameter creatinine was not significantly altered during ETI therapy.

ETI treatment did also evidently affect “inflammation markers”, as there was a clear decrease of peripheral leukocytes (WBC) as well as the levels of the immunoglobulins A, G and M. Contrarily, C-reactive protein does not show any relevant differences between study points as there were not any relevant elevations prior to treatment. Also, immunoglobulin E is not significantly altered during ETI therapy.

We also found evidence that the levels of HbA_1c_ decreased slightly during ETI treatment. The change did not differ between patients with CF-related diabetes or without. Unfortunately, in this study, the assessment of parameters specific for evaluating a pancreatic insufficiency was not included.

Subcategory analysis for age, CFTR modulator pretreatment, *Pseudomonas aeruginosa* status as well as genotype (homozygous F508del vs. heterozygous F508del) did not show any differences for neither of the mentioned parameters.

### 3.4 ETI treatment improves *Pseudomonas aeruginosa* (PsA) colonization status and protects from PsA colonization

**Fig. 2:**
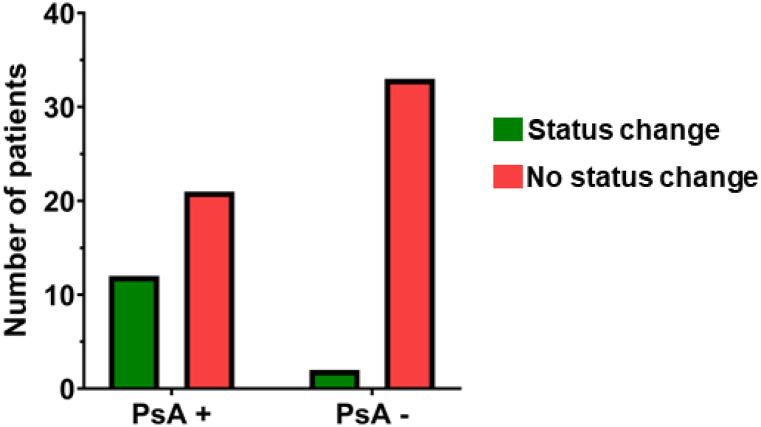
Proportion of *Pseudomonas aeruginosa* (PsA) status change after one year of ETI therapy.

Furthermore, we sought to determine whether we could detect any changes regarding the *Pseudomonas aeruginosa* (PsA) colonization status during the ETI therapy. At least one positive result for PsA (sputum or throat swab) in the microbiological testing was deemed to label a patient PsA positive. In our cohort, 15/69 patients received deep throat swabs prior to therapy. Out of these 15 patients, 13 patients were followed-up by throat swabs and two patients by sputum after six months of therapy. 52/69 patients were able to produce sputum prior to therapy. Under ETI treatment, 31 out of these 52 patients continued to produce sputum, whereas 21 patients had to be tested by throat swab. Most patients were already PsA positive at initiation of ETI treatment (57%) and thus displayed a rather chronic PsA colonization. Of note, we found a substantial number of PsA positive patients prior to ETI that sustainably displayed three or more negative results in the further course of the ETI treatment. In numbers, out of 33 PsA^+^ patients, 12 patients (36%) were considered PsA^−^ (Fig. 2, PsA^−^, green bar) after one year of ETI therapy. Among those 12 patients with PsA status conversion, PsA status was assessed by sputum culture in seven patients, whereas four patients received deep throat swabs. Only one patient was not able to expectorate sputum under ETI and thus received deep throat swabs in the therapy course.

In the other 22 PsA^+^ patients (64%), colonization status did not change (Fig. 2, PsA^+^, red bar). In contrast to that, only two patients out of 35 PsA^−^ patients (6%) at baseline were PsA colonized as confirmed by positive culture during the treatment (Fig. 2, PsA^−^, green bar). The remaining 33 patients (94%) stayed PsA^−^ throughout the therapy.

Statistical analysis using Fisher’s exact test of these numbers in a 2 × 2 table revealed an odds ratio of 8.8 (95% CI 1.9 to 40.9, p = 0.0038) for a favorable outcome, i.e., a change in PsA status from PsA positive to negative or remaining PsA negative under ETI treatment.

## 4 Discussion

The introduction of the CFTR-modulating triple therapy Elexacaftor – Tezacaftor – Ivacaftor represents a hallmark in the treatment of most patients with CF. Here, we now provide real-world data on the effectiveness of ETI therapy two years after market admission in Germany. The aim of our study was to systematically analyze therapy effects on clinical and laboratory parameters assessed in daily practice.

In the overall patient cohort, we can report major improvements regarding lung function: First and foremost a large increase in FEV_1_ by roughly 10%-points and also other lung function parameters such as MEF25, RV or sReff improve significantly. In patients with CF naïve to any kind of CFTR modulators other than ETI, the improvement in FEV_1_ even accounted for almost 14%-points. Therefore, our findings are nearly coherent with the phase 3 study of Middleton et al. [9] where 14.3%-points FEV_1_ increase following a 24 week ETI treatment interval has been reported in patients with CF with a F508del/minimal function phenotype. Our findings are also in line with the results of the PROMISE study comprising a rather large US-American CF patient cohort that have reported similar results from daily practice [15].

Moreover, levels of sweat chloride also decreased markedly underscoring the gain of function effect that the small molecules Elexacaftor, Tezacaftor and Ivacaftor exert on CFTR channel function [9,15].

Also, we can confirm the results of Middleton and Heijerman’s findings regarding an increase in BMI [9,10] as we could also show a relevant improvement by 0.71 points and 0.3 points for BMI Z-score respectively. The obvious assumption that ETI treatment may positively influence the metabolic status is further underlined by the improvements of patients' HbA_1c_ levels by −0,31 points. This result has also been confirmed by the reports of Petersen et al. [16].

We only detected mild to moderate elevations of liver enzymes, however, except for two patients these did not cross the threshold of a 5-fold increase of the upper limit, where ETI treatment is recommended to pause. However, after 24 weeks of therapy, there still was a significant elevation of ALT in the overall patient cohort compared to therapy initiation. Moreover, 15 out of 69 patients (21%) displayed elevated transaminases, a percentage twice as high as the findings reported in the approval study [9]. We also found a significant increase of both, direct and total bilirubin, whereas γ-GT as another cholestatic parameter did not show any relevant changes. Here, our findings stand contrarily to the reported results derived from a smaller, retrospective monocentric study where significant changes in AST and γ-GT have been described [17].

Regarding inflammation parameters, we can state that ETI treatment effectively normalizes systemic inflammation markers such as WBC or immunoglobulins A and G. These findings have also been shown for a CFTR-modulating therapy with Lumacaftor and Ivacaftor [18]. For ETI, this had not been reported yet. Also the slight increase of serum albumin of ∼3 g/dl can be seen in that context, as albumin has been shown to have a reciprocal relationship to the aforementioned inflammation markers and is also able to predict the severity of lung deterioration and systemic inflammation in the longitudinal course [19,20]. In cystic fibrosis, hyperinflammation has been shown to not only occur due to extrinsic factors like colonization with pathogenic germs but also as intrinsically caused by CFTR deficiency or dysfunction in immune cells itself [21,22]. Consequently, we postulate that the observed normalization of the inflammation parameters WBC, IgG and IgA as well as the Albumin/Globulin-ratio is at least partially due to a direct therapy effect on restoration or normalization of the immune system activity. This notion is further supported by the study of Favia et al. where the colleagues could show that Ivacaftor monotherapy is able to restore CFTR expression and function in mononuclear cells [23]. Also, treatment with Ivacaftor has been shown to restore proper immune cell responses like the formation of extracellular traps (NETs) in CF neutrophils [24] or phagocytic function against *Pseudomonas aeruginosa* in CF macrophages [25].

The beneficial effect of ETI treatment on systemic inflammation parameters is also reflected by our finding of a significant PsA status conversion in initially PsA^+^ patients within a year after therapy initiation. There are several studies that have described a relative reduction of PsA abundancy under a CFTR modulator treatment [[26], [27], [28]]. Moreover, Sosinski et al. also reported a decrease of PsA RNA quantity in sputum samples of patients with CF under ETI treatment [29].

Regarding our observed proportions of PsA status conversion under ETI treatment, our findings are in line with current literature as Heltshe et al. reported similar findings with a shift towards PsA^−^ cultures in ∼30% of initially PsA^+^ patients with G551D mutation and treated with Ivacaftor [30]. This effect may be ascribed to an increased bacterial clearance due to improved rheologic behavior of the airway surface liquid (ASL) [31], but also restored immune cellular functions like phagocytosis or a differentiation shift within the T helper cell compartment under CFTR modulator treatment [32,33] might to contribute to this effect. However, further functional studies are necessary to validate our findings.

Clearly, the presented study also displays some limitations: On the one hand, due to the monocentric study design and partially retrospective data analysis, only a limited number of patients could be enrolled with some data missing for some patients. Moreover, the heterogeneity of our study cohort must be considered which is why we have performed subcategory analyses for each potential confounder. Regarding PsA colonization status, results have to be interpreted with caution as results derived from sputum and to a smaller amount also from deep throat swab samples.

Taken together, we can summarize that a 24-week treatment interval with Elexacaftor, Tezacaftor and Ivacaftor effectively attenuates inflammatory parameters like WBC counts, immunoglobulins and albumin and improves lung function, BMI as well as sweat chloride. Another important finding is a promising decline of *Pseudomonas aeruginosa* colonization in our patient cohort. ETI treatment proved also relatively safe, however special attention and monitoring should be given to liver associated transaminases, bilirubin and γ-GT.

## Grant support

AH received funding support from the *Frieda-Marohn-Stiftung*.

## Author contributions

AS, JW and AH conceived and designed the study. RR, AG, CT, JS and EP contributed reagents, materials, analysis tools or data. HH, NK and AS performed the experiments, analyzed and interpreted the data. AS and AH wrote the manuscript.

## Declaration of competing interest

AS is stock owner of Vertex Pharmaceuticals. AH has received research grants for clinical studies, speaker’s fees, honoraria or travel expenses from Abbvie, Astellas, MSD, Novartis, Nutricia and Shire/Takeda.
